# Supplementary material for: Bilateral Germ Cell Tumor of the Testis: Biological and Clinical Implications for a Stem Versus Genetic Origin of Cancers
Source: Cells. 2025 Apr 30;14(9):658. doi: 10.3390/cells14090658 (PMC12071550; doi:10.3390/cells14090658)
Supplement: Supplementary file 1 [file cells-14-00658-s001.zip › cells-3522849-supplementary.pdf]

Bilateral Germ Cell Tumor of the Testis: Biological and Clinical Implications for a Stem Versus Genetic Origin of Cancers

Jamaal C. Jackson [1,†], Darren Sanchez [1,†], Aron Y. Joon [2], Marcos R. Estecio [3], Andrew C. Johns [4], Amishi Y. Shah [4], Matthew Campbell [4], John F. Ward [1], Louis L. Pisters [1], Charles C. Guo [5], Miao Zhang [5], Niki M. Zacharias [1,*] and Shi-Ming Tu [6,*]

1 Department of Urology, The University of Texas MD Anderson Cancer Center, Houston, TX 77030, USA; jjackson10@mdanderson.org (J.C.J.); dsanchez7@mdanderson.org (D.S.); jfward@mdanderson.org (J.F.W.); lpisters@mdanderson.org (L.L.P.)
2 Department of Biostatistics, The University of Texas MD Anderson Cancer Center, Houston TX 77030, USA; ayjoon@mdanderson.org
3 Department of Epigenetics and Molecular Carcinogenesis, The University of Texas MD Anderson Cancer Center, Houston, TX 77030, USA; mestecio@mdanderson.org
4 Department of Genitourinary Medical Oncology, The University of Texas MD Anderson Cancer Center, Houston TX 77030, USA; acjohns@mdanderson.org (A.C.J.); ayshah@mdanderson.org (A.Y.S.); mcampbell3@mdanderson.org (M.C.)
5 Department of Pathology, The University of Texas MD Anderson Cancer Center, Houston TX 77030, USA; ccguo@mdanderson.org (C.C.G.); mzhang8@mdanderson.org (M.Z.)
6 Division of Hematology/Oncology, University of Arkansas for Medical Sciences, Little Rock, AR 72205, USA
* Correspondence: nmzacharias@mdanderson.org (N.M.Z.), stu@uams.edu (S.-M.T.)
† These authors contributed equally to this manuscript.

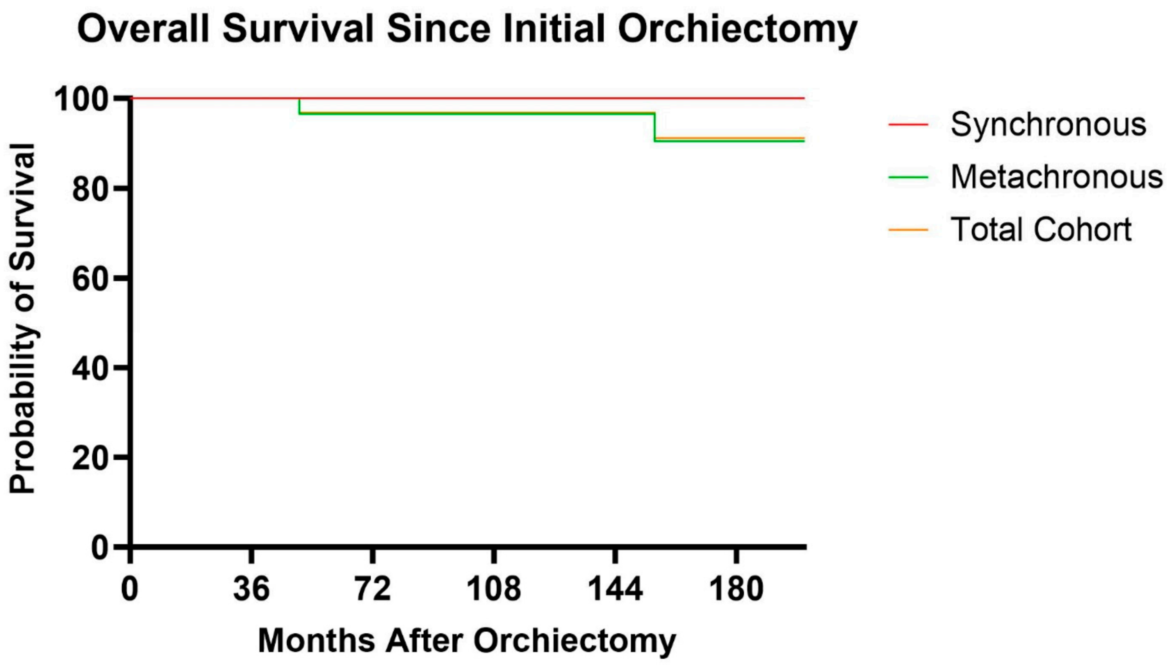

Supplemental Figure S1. Overall survival of patient cohort separated into synchronous (red line), metachronous (green line), and total cohort (yellow line). Log-rank p-value between groups is 0.66. Hazard ratio and median survival time were not calculated as one group had zero events.

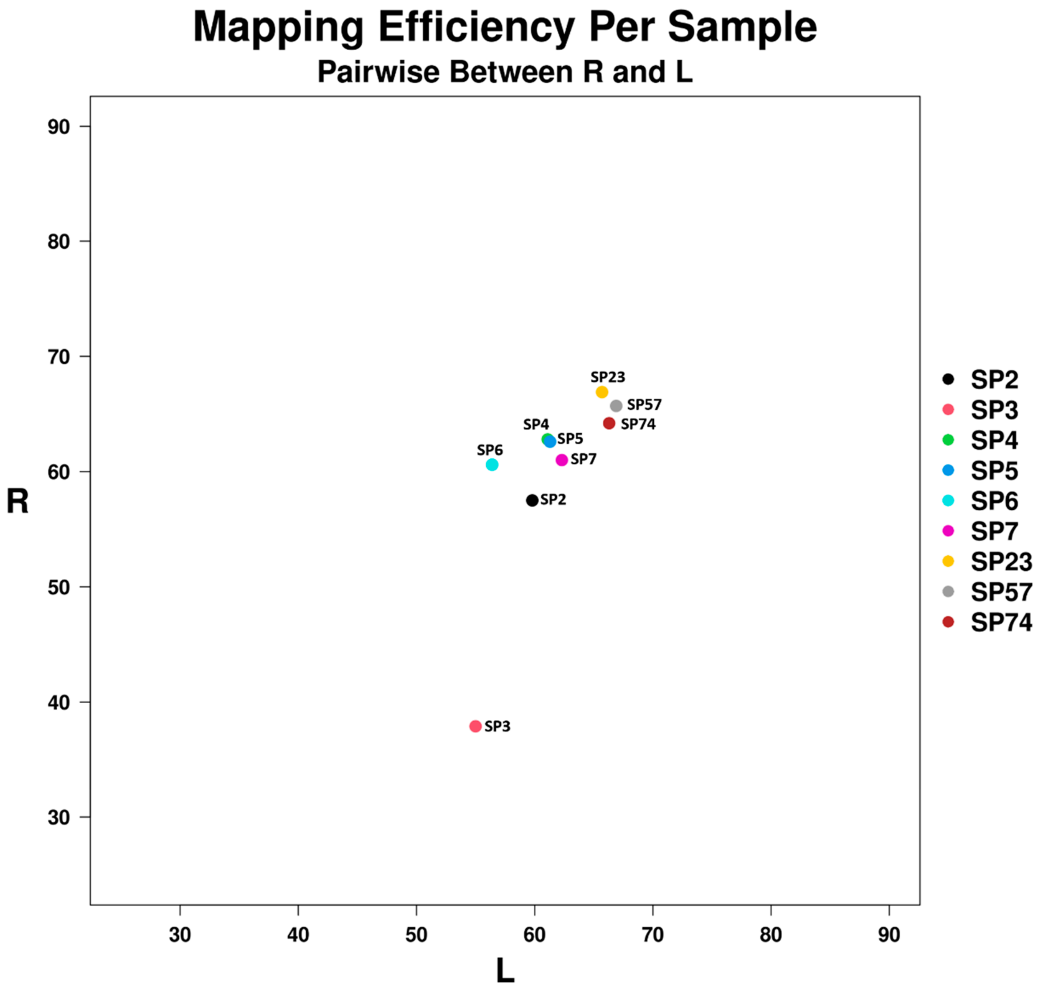

Supplemental Figure S2. DNA methylation mapping efficiency plotted pairwise between right and left GCT. The minimum coverage was 12.07, the maximum coverage was 28.72, with a median coverage of 17.71. For SP3, the coverage of SP3_L was16.67 and SP3_R was 13.43.
